# Hospital costs associated with surgical morbidity after elective colorectal procedures: a retrospective observational cohort study in 530 patients

**DOI:** 10.1186/1754-9493-8-2

**Published:** 2014-01-03

**Authors:** Evita Zoucas, Marie-Louise Lydrup

**Affiliations:** 1 Department of Surgery, Skåne University Hospital, Lund/Malmö, Sweden

**Keywords:** Elective colorectal procedures, Outcomes, Costs

## Abstract

**Background:**

Postoperative complications contribute to morbidity and mortality. This study assessed the impact of surgical complications on healthcare resource utilization for patients undergoing elective colorectal procedures.

**Method:**

Data were obtained on 530 consecutive colorectal operations performed from January 2010 to January 2011. Patient demographics, type of procedure, surgical complications classified as Clavien 1–5, length of stay, 60-day readmission rate, and hospital costs were recorded.

**Results:**

Seventy-five percent of the operations were associated with malignancy, and 26% were pelvic procedures. Thirty-five percent of the patients developed at least one complication, 21% of the complications did not require intervention. The readmission rate was 7.4%. Nine patients died during 60-day post discharge follow up.Median length of stay was 9 (3–34) days in uncomplicated and 16 (4–205) days in complicated cases. Occurrence of any complication at index admission increased total hospital costs 2.1-fold (EUR 25,680 vs. EUR 12,405), with the largest cost differential attributed to wound dehiscence and/or suture line failure requiring reoperation. These increases were primarily due to prolonged hospitalization and ICU expenditures. Readmission resulted in a further increase to an average cost of EUR 12,585 per re-admitted patient.Multivariate analysis showed that BMI > 25, obesity, operation complexity and surgeon significantly affected the risk for complication. Also, hospital costs were significantly increased by any postoperative complications, reoperations, high complexity of surgical procedures and high comorbidity index.

**Conclusions:**

Reducing morbidity after colorectal procedures improves quality of care and patient safety, and may also substantially reduce hospital costs and increase the efficiency of resource utilization.

## Introduction

Increasing public scrutiny of the quality of care provided by hospitals has prompted studies of the documentation, management, and prevention of complications [1-3]. In that context, safety and quality have become prominent criteria in the evaluation of surgical care. The incidence of postoperative complications in patients subjected to colorectal surgery has been shown to vary between 17% and 31% in investigations of both elective and emergent procedures [4,5]. In recent reports, postoperative complications increased health care costs and resource utilization [6,7]. In support of those observations, Soop et al. [8] noted that adverse effects were common and caused significant consumption of healthcare resources in Swedish hospitals, although hospital costs per se were not reported. Frequent peri-operative complications as infections and haemorrhage are potentially preventable, thus the control of cost in surgical patients may be inherently associated with provider outcomes.

The aim of the present review was to analyze the incidence, nature, and severity of postoperative complications after elective colorectal procedures performed at a tertiary care center, and to explore the association between surgical outcomes and hospital costs.

## Study population and methods

We conducted an observational retrospective cohort study at Skåne University Hospital, in southern Sweden. The hospital serves as a tertiary care referral center with a catchment of 2,1 miljon (approx. 1/5 of the total population). The colorectal unit is by case volume the largest in the country. Data from the operative database for elective colorectal procedures (ORBIT) was merged with the hospital internal accounting data and the hospital internal accounting database (FINN). All in- and out-patient records from January 2010 to April 2011 were systematically reviewed. All patients who underwent elective laparoscopic (5 cases) or open abdominal colorectal procedures during the period 1 January 2010 to 31 December 2010 were included. The patients were included in the unit’s standard enhanced recovery after surgery clinical care protocol which is based on the concensus protocol described by Fearon et al. [9]. Colorectal cases involving out-patient procedures during that period were excluded.

Clinical data on patient demographic characteristics, comorbid status, disease, and type of surgical procedure are shown in Table 1. The patients were stratified into four groups according to their body mass index (BMI), considering values > 25 to indicate overweight and > 30 obesity. Comorbid status was assessed using the Deyo-Charlson index [10]. Index surgical procedures were arbitrarily divided into four groups based on the complexity of each procedure. Operating time, blood loss, and attending surgeon (14 specialists in total) were noted, along with length of hospital stay, number and type of complications, unplanned return to surgery (reoperation), and readmission. Complications occurring during index admission until the patient was discharged or during 60-day post-operative follow-up for those discharged earlier were analyzed. The severity of each complication was classified according to Clavien et al. [11]. The most severe complications were those resulting in death (grade V). The severity of all other complications was defined by the morbidity inflicted. Complications were thus graded I (any deviation from normal postoperative course without the need for pharmacological treatment or intervention), II (requiring pharmacological treatment with eg antibiotics, b-blockers or blood tranfusions and total perenteral nutrition), IIIa (requiring surgical, endoscopic or radiological intervention without general aneasthesia), IIIb (intervention under general aneasthesia) and IV (life-threatening, ICU) (Table 2).

**Table 1:** Patient characteristics (n = 530)

Age, median (range)	68 (18–97)
Female gender, n (%)	251 (47.2)
BMI, n (%)	
> 20	39 (7.4)
20–25	280 (53.8)
26–30	158 (29.8)
> 30	52 (9,8)
Comorbidity index (Deyo-Charlson), median (range)	2 (0–7)
Malignancy, n (%)	397 (75)
Surgical complexity, n (%)	
(1) Small bowel, colon resection, colectomy	280 (52.8)
(2) Sigmoid, recto-sigmoid resection	81 (15.3)
(3) Rectum (resection, amputation)	144 (21.2)
(4) Multivisceral resection	25 (4.7)

**Table 2:** Outcome after colorectal surgery

**Outcome**	**N**	**Incidence (%)**
No complication	344	(64.9)
Any complication	186	(35.1)
Clavien grade I	2	
II	37	
IIIa	65	
IIIb	56	
IV	20	
60-day mortality	9	(1.7)
Surgery-related deaths	6	(1.1)
Reoperation	55	(10.4)
60-day readmission	41	(7.4)

Total hospital costs were regarded as estimated expenses accrued from indirect and direct patient care during a hospitalization period. Direct costs comprised all costs for providing the care, such as physician and nursing staff salaries and expenditures on medications and other medical supplies. Overhead costs included the financing of administrative infrastructure and fixed facilities.

The estimation of cost was based on the following units: the number of days for in-hospital care, total minutes under aneasthesia and total hours in the post- operative care unit or/and ICU. In the present system costs for salaries, medication and medical supplies were incorporated in the above units.

Total costs accrued during the index hospitalization period and during readmission were recorded separately. Costs for the two patients with Clavien grade I minor complications did not significantly affect total cost, while Clavien II complications although minor prolonged in-hospital care and thus affected costs. We analyzed expenditures on the following: nursing care in hospital wards, operative procedures, postoperative and intensive care units per index hospitalization period. Further subgroup analysis of costs was performed for the most common surgical complications.

### Statistical analysis

Descriptive statistics are shown as medians (interquartile range), means ± SE, and frequencies or percentages (95% Confidence interval) when appropriate. Comparisons of groups were carried out using Mann-Whitney’s test or Kruskal- Wallis test. Differences were considered significant if p > 0.05. Multivariate logistic regression was conducted when the response variable was the presence or absence of complication. The following model variables were pre-specified potential predictors: age, gender, BMI, comorbidity index, malignancy, complexity of procedure, and attending surgeon.SPSS (SPSS Inc.USA) software was used for the statistical analyses.

## Results

Five hundred thirty patients with a median age of 68 years underwent open or laparoscopic colorectal procedures during the 12-month study period. The majority of the patients were male, 29,8% were overweight and 9,8% were obese, but in general had few comorbid conditions. Three quarters of all surgical procedures were associated with malignancy, and 26% were pelvic procedures, which are considered to be major surgery (Table 1). One hundred eighty-six patients (35.1%) experienced at least one postoperative complication, and the majority of those conditions (76%) were classified as Clavien IIIa–IV . More than 40% of complications were severe (Clavien IIIb–IV), and 99% of all complications (Clavien II–IV) led to prolonged hospitalization. There were six surgery-related deaths, and a further three deaths occurred during the study period. The rates of reoperation and readmission were 10.4% and 7.4%, respectively (Table 2). The patients who were readmitted required further 465 days of hospital care at a total cost of EUR 515,899 (EUR 12,585/patient).

The most frequent complications were wound disruption (23) and superficial (30) or deep (28) wound infections. Failure of suture or staple line occurred in 21 of the 425 patients carrying such. One of the later cases was associated with a superficial wound infection and six with deep wound infections. Perioperative bleeding occurred in 13 cases. There were 32 urinary complications (including urinary tract infection and bladder dysfunction), 18 cardiovascular complications, two cases of pneumonia, two cases of graft necrosis, and five cases of central vein catheter sepsis. Bowel dysfunction or obstruction was noted in 18 patients following index procedure. Operation time for patients who developed complications and those without complications did not differ statistically.

Length of stay was prolonged by 78% following complications and was prolonged even further in cases involving wound disruption, suture line failure, deep abscesses, or return to the operating theater. The presence of complications led to substantially elevated hospital costs (Table 3). Incrementally, increases in the costs of ward care and ICU were greater than increases in operating room costs, representing longer care in surgical wards (days) or the ICU (hours) than length of operative procedure (minutes under aneaesthesia). Ward costs were raised 6- fold by failure of suture lines and 4,5-fold by wound dehiscence or deep wound infection, whereas increases in operation costs for those reasons were 2,5- and 1,5-fold, respectively (Table 4).

**Table 3:** Outcome, length of stay, and total costs by type of complication

**Outcome**	**N**	**Length of stay in days**	**Total costs (EUR)**
		**Median (range)**	**Mean + _ SE**
No complication	344	9 (3–34)	12410 ± 384
Any complication	186	16 *(4–205)	25680 ± 2289*
Wound disruption	23	23* (7–50)	29230 ± 3022*
Suture/Staple line dehiscence	21	30* (6–205)	47306 ±17194*
Superficial site infection	30	15,5* (4–43)	21406 ± 2497*
Deep wound infection	28	22.5* (6–8)	27145 ± 3118*
Reoperation	55	28* (6–205)	38050 ± 2427*
Surgery-related deaths	6	19* (5–52)	25102 ± 5985*

*p > 0.05 Mann–Whitney test.

**Table 4:** Analysis of costs by type of complication

	**No complication**	**Any complication**	**Wound disruption**	**Deep wound infection**	**Superficial wound infection**	**Suture line failure**
	**n = 346**	**n = 184**	**n = 23**	**n = 28**	**n = 30**	**n = 21**
	**EUR**	**EUR**	**EUR**	**EUR**	**EUR**	**EUR**
Ward care	3842 ± 118	9458 ± 1200*	17991 ± 8043*	16531 ± 6142*	6647 ± 642*	23586 ± 9270*
Surgery	5144 ± 125	8218 ± 467*	8385 ± 841*	8790 ± 932*	6723 ± 563*	13836 ± 3016*
Postoperative/ ICU	754 ± 66	1949 ± 264*	2061 ± 564*	1569 ± 338*	1310 ± 30*	2022 ± 398*

Values represent mean ± SE.

*p > 0.05 Mann–Whitney test.

After adjustment for age, gender, and preoperative comorbidity, multivariate analysis showed that surgical outcomes were independently associated with overweight (BMI > 25), obesity and high complexity of the surgical procedures (Table 5). Outcomes of individual surgeons were significantly related to the frequency of complications in the above multivariate analysis (p > 0,0083) with odds ratio for complications varying between 4,02 (95% confidence interval 1,59-10,10) and 0,51 (95% confidence interval 0,16-1,63). Statistical analysis of variance also demonstrated that accumulation of costs was correlated with the comorbidity index, the complexity of surgical procedures, the presence of complications, and the need for reoperation (Table 6). Moreover, complications remained associated with increased length of hospital stay and costs even after adjusting for surgical complexity.

**Table 5:** Risk factors affecting outcome of colorectal surgery

**Variable**	**p value**	**Odds ratio for complication**	**95% Confidence interval**
Gender: male vs. female	0.483	1.004	0.672–1.500
Age > 75 years	0.667	1.109	0.690–1.775
Comorbidity: high vs. low	0.360	0.936	0.811–1.079
BMI > 30	> 0.0005 ***	4.560	2.287-9.094
BMI 25–30	> 0.0005***	1.859	1.830–2.924
BMI 20–25		1,000	
BMI > 20	0.639	1.226	0.532–2.870
surgical complexity: high vs. low	> 0.00001***	1.826	1.449–2.302

***p> 0,001.

Multivariate logistic regression analysis.

**Table 6:** Accumulation of costs (EUR) in relation to comorbidity score (1-6+), complexity of procedure (1–4), presence of complication or re-operation

**Comorbidity score**	**N**	**Mean EUR**	**Std. Error**	**95% Confidence interval for Mean**		**P-value**	
				Lower bound	Upper bound		
0	132	12760	677	11420	14100		
1	41	28480	9340	9600	47360		
2	208	15970	862	14270	17670		
3	89	17220	1122	14990	19450		
4	22	2,600	3442	16440	30750	0,000003***	Kruskal-Wallis
5	15	16580	2166	11930	21230		
6	23	24770	4302	15850	33690		
Total	530	17060	884	15330	18800		
Operationcomplexity							
1	280	14810	1468	11920	17700		
2	81	15680	1086	13520	17850	0,000001***	Kruskal-Wallis
3	144	19700	1198	17340	22070		
4	25	31590	3039	25320	37870		
Total	530	17060	884	15330	18800		
Complication							
absent	344	12410	384	11650	13160		
present	186	25680	2289	21160	30200	0,000003***	Mann–Whitney
Total	530	17060	884	15330	18800		
Re-operation							
absent	475	12880	402	10950	13970		
present	55	38050	327	35380	42200	0,00001***	Mann–Whitney
Total	530	17060	884	15330	18800		

Multivariable analysis of means.

## Discussion

In the present review, we found that postoperative complications were common after elective colorectal procedures, occurring in 35.1% of the patients who underwent abdominal surgery. To our knowledge, this is the first Swedish study to demonstrate the direct correlation between surgical outcome and hospital costs in a consecutive series of elective colorectal operations. The most frequent complications recorded as surgical site infection, wound disruption, suture line failure as well as peri-operative haemorrhage might be considered provider related and potentially avoidable.

Elective colorectal surgery has been shown to have a low mortality rate but a high morbidity rate ranging from 9% to 31%. Notably, use of fast-track protocols in colorectal cases has been found to improve the outcome, seen as a 50% reduction in the number of patients with complications after surgery [12-14]. Despite a target length of stay of 3 to 5 days in fast-track protocols, such as the one applied currently, patients without complications in our series were hospitalized for a median of 9 days. Even though standard postoperative care routines were established, achieving continuous optimization of protocol adherence proved to be a challenge. Clearly, further prospective studies are needed to identify the rate of compliance with multimodal rehabilitation in the present unit.

Surgical site infection (SSI) and anastomotic leak have been described as the most common adverse events in several investigations [15-17]. Internationally, SSI is considered to be a major indicator of the quality of surgical care, and it has been estimated that 40% to 60% of SSIs are preventable [18]. The rates of superficial and deep wound infection noted in the present study are similar to data published by Hawn *et al.*[19] and Tang *et al.*[20], and compare favourably to values reported in several earlier publications [21-23]. Male sex, obesity, specific surgeons or hospitals, ostomy and total/subtotal colectomy were identified as risk factors for surgical site infection after colorectal surgery [22-25]. The circumscript number of cases present in our cohort did not allow subgroup analysis with regard to any technical aspects associated with SSI.

The incidence of anastomotic leak in elective cases has been found to vary between 1.4% and 7.9%, with the higher value noted in cases involving rectal procedures using an extraperitoneal suture line. Large series (> 800 patients) reported overall anastomotic leak rate under 4% [17,20,24,26].

Moreover, the risk of anastomotic leak has been observed to be increased by long operation time, low level of colorectal anastomosis, and comorbid conditions [27,28]. The absence of consensus regarding the definition or the diagnosis of anastomotic leak makes it difficult to compare the rates of anastomotic failure published in the literature. Here, we considered dehiscence of the suture line in a blind colon or rectal segment to be similar to an anastomotic leak in terms of morbidity, and we included patients with disruption of the distal stump after Hartmann’s procedure in this group. Both clinical and radiologic anastomotic leaks were registered. Yet, it is probable that the number of radiologic anastomotic leaks was underestimated as asymptomatic patients did not undergo routine radiologic investigation post-operatively.

The incidence of midline wound disruption in our investigation was high compared to rates of 0–2.7% after elective procedures in contemporary studies [5,17,29]. Suture technique monitored by the ratio of suture length to wound length (4–6/1) has proven to be the most important factor affecting this variability [29], which indicates that midline dehiscence could easily be rectified by dissemination of appropriate knowledge and adherence to evidence-based techniques.

Our results showed that age did not increase the risk of complications, which is contrary to previous findings and could be attributed to the fact that only elective cases were included [5,17]. Large studies have shown that > 10% weight loss prior to surgery and overweight constituted independent risk factors for morbidity after colorectal surgery [16,30]. As in the present report, several investigations [16,25,30,31] recognised that BMI above 25 and obesity were associated with increased risk for surgical complications. Complex procedures were also correlated with higher complication risks in our study, as has been reported by other researchers [32]. The rate of re-operation after colorectal surgery was deemed to be an independent factor for surgical quality. In the present review this factor compared favourably with contemporary studies [33].

The attending surgeon was also found to be an independent risk factor for complications in a fixed-variable statistical model. We regarded this model most appropriate, because the surgeons in the present cases were not chosen at random, but were instead assigned to specific operating lists according to their knowledge, training, and experience. In agreement with our findings, these aspects of surgeons have been found to have an impact on surgical outcome in numerous other studies [20,34,35]. Drolet *et al.*[36] observed decreased morbidity and mortality in patients who underwent elective colorectal procedures performed by experienced surgeons, and Hubner and colleagues [22] noted that the surgeon constituted an independent risk factor for SSI after colon surgery. Surgical skills were hard to assess as the large number of assessment methods indicated [37]. Yet, these skills were recognized as essential in the occurrence of complications [38]. We identified a large difference in the odds ratio for complication between individuals which indicated a distinct disparity within this group of surgeons.

Major part of the adverse events (wound infection, anastomotic failure, wound dehiscence, and perioperative bleeding) observed in our patients was avoidable, which is in accordance with contemporary studies [4,8]. These reports also found technical error to be the most common error type in surgery. Presumably, some patients will always develop such complications, but, for any given patient, that should not be an expected outcome. It is also obvious, that, once identified, avoidable adverse outcomes, should be readily amenable to rectification.

Our analysis showed that postoperative complications were associated with a substantial and statistically significant increase in total hospital costs, even after adjustment for type of surgery and comorbid conditions. The relative increase ranged from 207% for any complication to 380% for suture line failures, and, concomitantly, length of hospital stay was significantly prolonged.

Nursing and postoperative or intensive care costs made the main contribution in this context, whereas the contribution from surgical operation costs was smaller than we had expected. Expenditures related to reoperations during index hospitalization,per se, could not be retrieved separately from the database we used. Costs were further increased by readmissions, even if the currently observed readmission rate of 7.4% compares favorably with results obtained in earlier studies [39,40].

These results were consistent with findings by other investigators who have demonstrated a linear correlation between surgical outcomes and accrued hospital costs. Robust evidence showed that programs for continuous quality improvement in surgical care, based on measurement and monitoring of outcome- and process- based quality indicators, were effective in reducing post-operative morbidity, mortality and hospital costs [6,41-43]. In their analysis of 676 US hospitals, Fry et al. [43], indexed outcomes and efficiency to a reference set of cost effective hospitals and were able to demonstrate that inefficiency was the major contributor to excess cost. Colorectal procedures in the study population had a complication rate of 7, 7% in index and 16,3% in inefficient sites which resulted in 100% cost increase per incurred complication, a level that is significantly lower than the corresponding rates noted in the present review. As we identified that adverse outcomes resulted in very high average costs per event, it is clear that important cost savings could be achieved by reducing the occurrence of complications.

The present observational cohort study had a number of limitations. The data were derived retrospectively from consecutive patients treated at a single tertiary care unit during 2010, and hence the results might not be applicable to other institutions or other time periods. In an attempt to eliminate the inaccuracy of administrative data in identifying complications, we conducted a systematic review of the surgical records of all in- and out-patients in the index population and collected information on operations from the surgery-specific database. Unfortunately, the surgical unit did not have a structured post-discharge surveillance program for SSIs, and thus there was probably an underestimation bias. Outcomes for discharged patients were evaluated 60 days postoperatively, and therefore long-term outcomes such as incisional hernias and aberrations in urinary and intestinal functions were not registered. The hospital internal accounting database was based on estimates correlated to diagnosis and did not permit detailed estimates of expenditure such as hourly physician cost or medication and medical supplies cost per patient. Nevertheless, the deduction that postoperative complications are independently related to high hospital costs and prolonged hospital stay remains true.

In conclusion, potentially avoidable complications following elective colorectal surgery were frequent in the tertiary care unit we studied, and this resulted in a considerable increase in hospital costs and resource expenditures. Focus on the improvement of surgical outcomes contributes substantially to better quality of care and patient safety and facilitates effective cost containment and resource utilization in healthcare.

## Competing interests

The authors have no conflicts of interest in regard to this manuscript.

## Authors’ contributions

EZMD, PhD and M-LL MD, PhD have both participated in the planning of the study, the collection of relevant data and preparation of the manuscript. Both authors read and approved the final manuscript.
